# Awareness, Treatment, and Control of Hypertension and Hypercholesterolemia Among Insured Residents of New York City, 2004

**Published:** 2011-08-15

**Authors:** Quynh C. Nguyen, Elizabeth Needham Waddell, Bonnie D. Kerker, R. Charon Gwynn, James C. Thomas, Sara L. Huston

**Affiliations:** Department of Epidemiology, University of North Carolina Gillings School of Global Public Health; Mailman School of Public Health, Columbia University, New York, New York. At the time of the study, Dr Waddell was affiliated with the New York City Department of Health and Mental Hygiene; New York City Department of Health and Mental Hygiene, New York, New York; Mailman School of Public Health, Columbia University, New York, New York. At the time of the study, Dr Gwynn was affiliated with the New York City Department of Health and Mental Hygiene; University of North Carolina Gillings School of Global Public Health, Chapel Hill, North Carolina; Maine Center for Disease Control and Prevention, Augusta, Maine, and University of Southern Maine, Portland, Maine. At the time of the study, Dr Huston was affiliated with the University of North Carolina Gillings School of Global Public Health

## Abstract

**Introduction:**

Health care access and sociodemographic characteristics may influence chronic disease management even among adults who have health insurance. The objective of this study was to examine awareness, treatment, and control of hypertension and hypercholesterolemia, by health care access and sociodemographic characteristics, among insured adults in New York City.

**Methods:**

Using data from the 2004 New York City Health and Nutrition Examination Survey, we investigated inequalities in the diagnosis and management of hypertension and hypercholesterolemia among insured adults aged 20 to 64 years (n = 1,334). We assessed differences in insurance type (public, private) and routine place of care (yes, no), by sociodemographic characteristics.

**Results:**

One in 10 participants with hypertension and 3 in 10 with hypercholesterolemia were unaware and untreated. Having a routine place of care was associated with treatment and control of hypertension and with awareness, treatment, and control of hypercholesterolemia, after adjusting for insurance type, age, sex, race/ethnicity, foreign birth, income, and education. Differences in systolic blood pressure and total cholesterol between people with versus without a routine place of care were 2 to 3 times the difference found between people with public versus private insurance. Few differences were associated with sociodemographic characteristics after adjusting for routine place of care and insurance type; however, male sex, younger age, Asian race, and foreign birth with short-term US residence reduced the odds of having a routine place of care. Neither income nor education predicted having a routine place of care.

**Conclusion:**

Sociodemographic characteristics may influence chronic disease management among the insured through health care access factors such as having a routine place of care.

## Introduction

In the United States, inequalities in health insurance coverage are well documented; for example, 12% of nonelderly non-Hispanic whites were uninsured compared with 34% of Hispanics (any race) in 2007 (1). Lack of health insurance has been linked to lower awareness, treatment, and control of hypertension and hypercholesterolemia (2) and with unmet needs for clinically indicated preventive services such as blood pressure and cholesterol screening (3-5). Moreover, clear sociodemographic inequalities in the management of hypertension and hypercholesterolemia have been identified (6-8). Using 2008 data from the National Health and Nutrition Examination Survey (NHANES), Egan and colleagues reported worse hypertension control among younger adults compared with middle-aged or elderly adults and among Hispanics (any race) compared with non-Hispanic whites (9). Nelson and colleagues found worse treatment rates for hypercholesterolemia among non-Hispanic blacks and Mexican Americans compared with non-Hispanic whites (7).

An investigation of insured adults that assesses potential associations of chronic disease management with both health care access and sociodemographic characteristics is required to better understand the sociodemographic and access inequalities that may persist after accounting for health insurance. A local-level analysis of New York City adults, a population that is largely insured but with higher proportions of racial/ethnic minorities and low-income people compared with the nation as a whole, is uniquely suited to identify inequalities among traditionally underserved populations and may be useful to other jurisdictions with similar populations. This study focuses on hypertension and hypercholesterolemia, 2 common behaviorally and pharmacologically modifiable cardiovascular disease risk factors. The main objective of this study was to examine awareness, treatment, and control of hypertension and hypercholesterolemia, by sociodemographic and health care access characteristics, among insured adults aged 20 to 64 years in New York City. The secondary objective was to assess potential associations of sociodemographic characteristics with insurance type and access to a routine place of care in this population.

## Methods

### Study sample

We used data from the 2004 New York City Health and Nutrition Examination Survey (NYC HANES), a population-based, cross-sectional survey of noninstitutionalized New York City adults aged 20 years or older. NYC HANES survey instruments, protocols, equipment, and measurements followed NHANES specifications. Detailed information on data collection components, protocols, and study design has been published elsewhere (10). In brief, participants were selected from June through December 2004 via a 3-stage sampling design. The survey included computer-assisted interviews, a physical examination, and laboratory testing. Of the 4,026 households approached, 3,388 households agreed to participate (88% response rate). Of the 3,047 eligible participants identified within households, 1,999 completed both the face-to-face interview and at least 1 comprehensive examination measurement (66% cooperation rate), for an overall response rate of 55% (10).

Most US adults aged 65 years or older are covered by Medicare insurance, so the health care access and sociodemographic factors that influence their chronic disease care may be substantially different from factors that influence the care of nonelderly adults (11). Therefore, we restricted the study population to participants aged 20 to 64 years. The final sample consisted of 1,334 insured adults. NYC HANES was approved by the New York City Department of Health and Mental Hygiene's institutional review board.

### Variable definitions

We combined self-reported race/ethnicity into the following classifications: non-Hispanic white, non-Hispanic black, non-Hispanic Asian, and Hispanic of any race. We recoded multiracial participants who selected a main race into the participant's selected category. We classified participants whose answers did not correspond to any of the 4 categories as "other," and estimates for this group are not reported because of small sample size (n = 28). We categorized age into 2 groups: 20 to 39 and 40 to 64 years. We defined participants born in the United States, including US territories (eg, Puerto Rico, Guam) as US-born. We considered length of stay in the United States as a proxy for other factors, including acculturation and familiarity with the US health care system, and categorized foreign-born respondents by time living in the United States (≥10 y, <10 y). We dichotomized annual family income as at least $20,000 and less than $20,000, which approximated the national poverty threshold in 2004 for a family of 4 (12). We defined education as a 3-category variable (no high school or some high school, but no diploma; high school diploma or equivalent; more than high school). We included examination-based measures of body mass index (kg/m^2^) as a continuous variable after log-likelihood ratio tests revealed that the addition of a quadratic term did not significantly improve model fit.

We examined health care access in the following 2 ways: insurance type (public, private) and having a routine place of care (yes, no). Insured respondents reported up to 2 health insurance plans. We classified insurance type as 1) any private health insurance or single-service plans, such as CHAMPUS/TRICARE or Veterans Affairs or 2) only Medicaid or other public health insurance. To define routine place of care, respondents were asked, “Is there a place that you usually go to when you are sick or you need advice about your health?” Using a standard definition (13), we categorized participants who indicated that they had 1 or more regular places to go to for their health care needs as having a routine place of care. Among those who had a routine place of care, less than 1% reported having more than 1 routine place of care. We examined reported number of medical visits in the previous 12 months as a potential mediator of the relationship between having a routine place of care and adequacy of disease management.

We defined hypertension (n = 212) as an average systolic blood pressure of at least 140 mm Hg or an average diastolic blood pressure of at least 90 mm Hg or self-report of taking prescribed medication for hypertension (14,15). Three to 4 blood pressure measurements were taken for each participant using standardized NHANES procedures and instruments (16). The average of blood pressure measurements was recorded, excluding the first measurement. If only 1 measurement was taken, it was used.

We defined hypercholesterolemia (n = 240) as having a measured serum total cholesterol (fasting or nonfasting) of at least 240 mg/dL or self-report of taking prescribed cholesterol-lowering medication (14,15).

We considered study participants to be aware of their hypertension or hypercholesterolemia if they had been told by a health care professional they had hypertension or that their blood cholesterol was high, respectively, and to be treated if they reported currently taking prescribed medication for their condition. Among those aware, we assessed level of control of hypertension by using measured systolic blood pressure and level of control of hypercholesterolemia by using measured total cholesterol.

### Statistical analysis

Using cross-tabulations, we first estimated the prevalence of health care access characteristics (insurance type, routine place of care) by sociodemographic characteristics. We used multiple logistic regression to assess having a routine place of care by sociodemographic characteristics (age, sex, race/ethnicity, nativity, income, education). We then compared the prevalence, awareness, treatment, and control of hypertension and hypercholesterolemia among the publicly insured and the privately insured (reference group). Statistical significance was set at *P* ≤ .05 and was determined by using 2-sided *t* tests of pairwise comparisons.

The final regression models of awareness, treatment, and control of hypertension and hypercholesterolemia among insured adults included both sociodemographic characteristics and health care access characteristics. We selected covariates based on a review of the literature and retained them in the models regardless of statistical significance (6-9). We examined inequalities in treatment and control of these conditions among participants who were aware of their diagnosis. We ascertained variation in levels of control in terms of mean systolic blood pressure and mean total cholesterol via multiple linear regression.

We conducted all analyses using SAS-callable SUDAAN version 10.0 (Research Triangle Institute, Research Triangle Park, North Carolina). We used weighted analyses to account for the complex survey design and further adjusted for component and item nonresponse. All summary statistics were weighted to produce population-based estimates, allowing for inferences to 3.7 million insured New York City residents aged 20 to 64 years.

## Results

More than 8 in 10 insured adults had a routine place of care (Table 1). Men, non-Hispanic Asians, adults aged 20 to 39 years, and foreign-born adults who had been in the United States fewer than 10 years were less likely to have a routine place of care. Racial/ethnic minority groups (vs non-Hispanic whites), foreign-born (vs US-born) participants, and lower income and education groups had higher proportions with public insurance.

The prevalence of hypertension was higher among publicly insured (22.3%) than privately insured participants (14.7%) (Table 2). Among people who were aware of their hypertension, publicly insured people had higher systolic blood pressure. However, treatment rates for hypercholesterolemia were higher among participants with public insurance (76.1%) than private insurance (53.2%).

Among people with hypertension, 88.9% (95% confidence interval [CI], 84.1%-92.3%) were aware of their condition. Participants without a routine place of care had lower odds of treatment and higher systolic blood pressure than those with a routine place of care (Table 3). Awareness of hypertension was lower among women (vs men), people aged 20 to 39 years (vs those aged 40-64 y), and those with less than a high school education (vs those with more than a high school education). Compared with private insurance, public insurance was associated with lower levels of hypertension control.

Among participants with hypercholesterolemia, 70.8% (95% CI, 63.6%-77.0%) were aware of their condition. Insured adults without a routine place of care had lower odds of awareness of hypercholesterolemia, lower odds of treatment, and higher total cholesterol than those with a routine place of care (Table 4). Public health insurance (vs private insurance) was associated with higher treatment rates for hypercholesterolemia. Hypercholesterolemia awareness, treatment, and control were worse for adults aged 20 to 39 years compared with adults aged 40 to 64 years. Annual family income of less than $20,000 was linked to lower awareness of hypercholesterolemia.

We examined the number of medical visits as a mediator of the relationship between routine place of care and disease management. After controlling for the number of medical visits within the past 12 months, lacking a routine place of care was no longer related to the treatment of hypertension or hypercholesterolemia, but it remained predictive of worse control of hypertension (β, 16.8; SE, 7.3) and hypercholesterolemia (β, 28.7; SE, 12.1) and lower awareness of hypercholesterolemia (adjusted odds ratio, 0.1; 95% CI, 0.1-0.4) (data not shown).

## Discussion

Inequalities in the medical management of hypertension and hypercholesterolemia go beyond health insurance (17,18). Among insured New York City residents aged 20 to 64 years, 1 in 10 with hypertension and 3 in 10 with hypercholesterolemia were unaware and untreated. Having a routine place of care was the most consistent predictor of hypertension treatment and control and was strongly linked to awareness and treatment of hypercholesterolemia and total cholesterol, even after adjusting for insurance type and sociodemographic characteristics. The differences in systolic blood pressure and total cholesterol between people with and without a routine place of care were 2 to 3 times those found between insurance type categories.

A previous NYC HANES study of adults aged 20 to 64 years with treated hypertension found that non-Hispanic blacks had fourfold lower odds of having their hypertension controlled compared with non-Hispanic whites (14). In our study of insured adults in the same age group, we did not observe racial/ethnic disparities in hypertension control among those aware or among those treated.

Further research is needed to elucidate reasons for not having a routine place of care among people with chronic conditions and the mechanisms through which lack of a routine place of care hinders disease management. We found that, although income and education were associated with having private health insurance, they were unrelated to having a routine place of care. Consistent with our findings, Viera and colleagues found that younger age and male sex were associated with lack of a routine place of care at the national level (13). Not having a routine place of care may be associated with a certain set of health beliefs or knowledge about prevention and disease management. Viera and colleagues found that two-thirds of adults said they did not have a routine place of care because they were seldom or never sick (13). Because hypercholesterolemia and other cardiovascular disease risk factors can often occur without symptoms, detection of these health conditions among otherwise healthy people during a routine physical examination may require a personal belief in the importance of preventive care. Moreover, patients' beliefs about the seriousness of hypertension, the root cause of hypertension, and self-efficacy in taking hypertension medications have been linked to hypertension control (19). Hence, because of their influence on patients' decision making, health beliefs and attitudes necessitate special consideration in efforts to improve chronic disease care.

Having a routine place of care has been linked to timely diagnosis and better management of hypertension and hypercholesterolemia (14,17). People without a routine place of care or a usual-care physician receive fewer preventive services, including blood pressure and cholesterol screening (20). Having a routine place of care may facilitate stronger patient-physician relationships and intensity of care, thereby improving disease management. DeVoe and colleagues found that people with a usual source of care were more likely to perceive positive health care interactions (eg, provider always listened to them) (21). In this study, after accounting for differences in the number of medical visits (22), having a routine place of care was unrelated to treatment for hypertension and hypercholesterolemia — suggesting that intensity of care may be one of the main mechanisms by which routine place of care influences treatment rates. Nonetheless, differences in awareness and control by routine place of care were only slightly attenuated by the inclusion of number of visits, suggesting the importance of other mediators in the relationship between routine place of care and disease management.

Although this study focused on hypertension and hypercholesterolemia, other chronic conditions such as diabetes, the prevalence of which is 12.5% in New York City, are also serious public health issues and underscore the need to implement programs and policies to reduce the burden of chronic conditions (23). In addition to the variables that we examined, additional factors, such as health literacy, processes of care (eg, racial/ethnic inequalities in therapy intensification), medication adherence, language barriers, and cultural beliefs, are also relevant to the diagnosis and control of illness (19,22,24-26). Area-level policies that promote healthy lifestyles can help prevent chronic conditions (26). Examples of policies introduced in New York City include increasing local cigarette taxes, restricting the use of artificial trans fats in restaurants, and increasing the number of mobile vendors that sell vegetables (27).

Our analysis was conducted on a population-based sample of New York City adults, and measurement error was minimized by using standardized quality assurance measures (15). The diversity of the NYC HANES sample permitted us to calculate reliable estimates among Asians and Hispanics, which are not available through NHANES, that allow for generalization of findings to other urban municipalities with large foreign-born and racial/ethnic minority groups. Disease management definitions incorporated information from both self-report and clinical data and hence allowed the inclusion of undiagnosed participants. This permitted an objective investigation of inequalities in both diagnosed and undiagnosed chronic conditions.

However, because NYC HANES sampled only noninstitutionalized adults, people in nursing homes and other institutions or group quarters were not surveyed. To address the potential selection bias due to the 55% survey response rate, analytic weights were adjusted for age group, race/ethnicity, and sex. Our definition of hypertension, which relied on average blood pressure measurements taken at 1 clinic visit, deviated from clinical practice guidelines for the diagnosis of hypertension, which is based on 2 or more clinic visits (28), but our assessment of inequalities in the clinical management of hypertension would be unbiased unless differences in the elevation of blood pressure at the clinic examination were systematic. Sample-size considerations led us to assess hypercholesterolemia by using total cholesterol rather than fasting low-density lipoprotein (LDL) levels, although treatment and control guidelines are based on the latter. Nonetheless, total cholesterol and LDL cholesterol categories similarly predict coronary heart disease risk (29). Moreover, small sample sizes also limited the precision of our estimates (eg, the potential effects of having a routine place of care).

The observed sociodemographic inequalities associated with having a routine place of care and the relationship between routine place of care and improved treatment and control of hypertension and hypercholesterolemia justify closer examination of prevention efforts targeted to traditionally underserved groups younger than 65 years, regardless of insurance status. Outreach to both medical care consumers and providers to promote the importance of having a medical home may be an effective strategy for improving chronic disease diagnosis and management, and for reducing inequalities in health care. Given the variety of reasons that people lack a routine place of care, efforts to help people have a routine place of care should be comprehensive in their approach — addressing financial barriers, access to primary care providers and to cardiology specialists (particularly at federally qualified community health centers), availability of appointments, patient perceptions about the necessity of a routine place of care, and insurance coverage (13,18,30). Increasing the uptake of routine places of care across all sociodemographic groups may improve overall chronic disease care among the insured.

## Figures and Tables

**Table 1:** Health Care Access Among Insured Adults Aged 20 to 64 Years, 2004 New York City Health and Nutrition Examination Survey

Characteristic	n^a^	Has Public Insurance		Has Routine Place of Care		

		% (95% CI)	*P* Value^b^	% (95% CI)	*P* Value^b^	AOR (95% CI)
All	1,334	29.3 (25.3-33.7)	NA	83.4 (80.7-85.8)	NA	NA
**Insurance type**						
Public	442	NA	NA	82.7 (79.7-85.4)	.25	1.5 (1.0-2.3)
Private	892	NA	NA	85.0 (81.0-88.3)	Reference	1 [Reference]
**Sex**						
Male	495	22.3 (17.9-27.4)	<.001	79.9 (75.4-83.8)	.01	0.7 (0.5-0.9)
Female	839	34.4 (29.4-39.7)	Reference	85.9 (83.0-88.4)	Reference	1 [Reference]
**Race/ethnicity**						
Non-Hispanic white	448	11.6 (8.6-15.5)	Reference	85.0 (80.5-88.6)	Reference	1 [Reference]
Non-Hispanic black	300	33.8 (26.7-41.7)	<.001	88.7 (84.7-91.8)	.16	1.3 (0.8-2.2)
Non-Hispanic Asian	168	45.6 (36.4-55.1)	<.001	74.1 (65.2-81.4)	.01	0.5 (0.3-0.9)
Hispanic	399	46.7 (41.3-52.3)	<.001	79.8 (74.7-84.0)	.08	0.6 (0.4-1.0)
**Age, y**						
20-39	659	27.1 (22.4-32.4)	.21	77.4 (73.7-80.8)	<.001	0.4 (0.3-0.6)
40-64	675	31.2 (26.2-36.7)	Reference	88.4 (85.1-91.1)	Reference	1 [Reference]
**Country of birth**						
US-born	740	23.6 (19.0-29.0)	Reference	85.2 (82.0-87.9)	Reference	1 [Reference]
Foreign-born, in US <10 y	188	44.7 (35.6-54.1)	<.001	69.5 (60.9-76.9)	<.001	0.5 (0.3-0.9)
Foreign-born, in US ≥10 y	395	32.7 (27.3-38.5)	.01	86.1 (81.9-89.4)	.74	1.2 (0.7-1.9)
**Annual household income, $**						
<20,000	371	78.3 (73.1-82.6)	<.001	83.7 (79.2-87.4)	.95	1.0 (0.7-1.5)
≥20,000	943	12.5 (10.3-15.1)	Reference	83.6 (80.8-86.3)	Reference	1 [Reference]
**Education level**						
<High school	313	62.3 (55.1-69.0)	<.001	83.2 (78.5-87.0)	.99	0.8 (0.5-1.3)
High school diploma or equivalent	241	35.1 (28.7-42.1)	<.001	85.3 (80.1-89.3)	.41	1.1 (0.7-1.6)
>High school	777	16.2 (13.1-19.9)	Reference	83.1 (79.7-86.1)	Reference	1 [Reference]

Abbreviations: CI, confidence interval; AOR, adjusted odds ratio; NA, not assessed.

a Sample size varies with missing data on covariates.

b Calculated by using *t* tests.

**Table 2:** Prevalence, Awareness, Treatment, and Control of Hypertension and Hypercholesterolemia, by Insurance Type, Among Adults Aged 20 to 64 Years, 2004 New York City Health and Nutrition Examination Survey^a^

Indicator	Publicly Insured		Privately Insured		*P* value^c^

	n	% (95% CI)^b^	n	% (95% CI)^b^	
**Prevalence**					
Hypertension	89	22.3 (18.4-26.9)	123	14.7 (12.1-17.6)	.003
Hypercholesterolemia	92	25.4 (20.8-30.6)	148	21.8 (18.5-25.4)	.23
**Awareness^d^**					
Hypertension	79	89.8 (81.7-94.6)	107	88.1 (80.9-92.8)	.71
Hypercholesterolemia	64	71.6 (61.3-80.1)	104	70.9 (61.8-78.6)	.90
**Treatment among aware^e^**					
Hypertension	70	87.9 (78.0-93.7)	91	85.6 (76.9-91.3)	.67
Hypercholesterolemia	48	76.1 (63.8-85.1)	54	53.2 (43.2-63.0)	.003
**Control among aware**					
Systolic blood pressure, mean (SD), mm Hg	79	135.0 (21.2)	107	128.9 (17.0)	.04
Total cholesterol, mean (SD), mg/dL	64	224.4 (53.2)	104	232.5 (42.4)	.30

Abbreviations: CI, confidence interval; SD, standard deviation.

a Sample sizes were unweighted; percentages and 95% CIs were weighted.

b Values are expressed as percentage (95% CI) unless otherwise indicated.

c Calculated by using *t* tests.

d Participants were considered aware if they been told by a health care professional that they had the condition.

e Participants were considered treated if they reported currently taking prescribed medication for their condition.

**Table 3:** Odds of Awareness, Treatment, and Control of Hypertension Among Insured Adults Aged 20 to 64 Years With Hypertension (n = 212), 2004 New York City Health and Nutrition Examination Survey^a^

Characteristic	Awareness,^b^ AOR (95% CI)	Treatment Among Aware,^b^ AOR (95% CI)	Systolic Blood Pressure Among Aware^c^	

			β (SE)	*P* Value
**Routine place of care**				
No	1.0 (0.2-5.6)	0.2 (0.1-0.8)	16.4 (6.9)	.02
Yes	1 [Reference]		0	Reference
**Insurance type**				
Public	1.2 (0.4-4.1)	1.1 (0.4-3.6)	6.7 (3.2)	.04
Private	1 [Reference]		0	Reference
**Age, y**				
20-39	0.2 (0.1-0.5)	0.5 (0.1-2.1)	−7.0 (4.3)	.11
40-64	1 [Reference]		0	Reference
**Sex**				
Male	3.7 (1.2-11.3)	0.4 (0.1-1.6)	4.5 (3.5)	.21
Female	1 [Reference]		0	Reference
**Race/ethnicity**				
Non-Hispanic white	1 [Reference]		0	Reference
Non-Hispanic black	1.6 (0.4-6.8)	1.5 (0.4-6.2)	4.1 (4.1)	.32
Non-Hispanic Asian	1.2 (0.2-7.3)	NC^d^	−4.0 (7.9)	.62
Hispanic	1.6 (0.4-6.7)	1.4 (0.3-7.8)	0.9 (4.6)	.85
**Country of birth**				
Foreign-born, in US <10 y	1.5 (0.3-8.2)	NC^d^	4.7 (4.7)	.33
Foreign-born, in US ≥10 y	0.9 (0.3-2.3)	0.7 (0.2-2.3)	3.8 (3.6)	.30
US-born	1 [Reference]		0	Reference
**Annual household income, $**				
<20,000	1.6 (0.4-6.5)	0.5 (0.1-3.2)	0 (3.0)	.99
≥20,000	1 [Reference]		0	Reference
**Education level**				
<High school	0.3 (0.1-1.0)	1.7 (0.3-8.9)	−0.9 (4.1)	.82
High school or equivalent	1.4 (0.2-8.3)	0.4 (0.1-1.6)	2.4 (3.7)	.53
>High school	1 [Reference]		0	Reference
**BMI, kg/m^2^**	1.0 (1.0-1.1)	1.1 (1.0-1.2)	−0.1 (0.2)	.77

Abbreviations: AOR, adjusted odds ratio; CI, confidence interval; SE, standard error; NC, not calculated; BMI, body mass index.

a All statistics were weighted.

b Calculated by using multivariate logistic regression adjusting for all covariates listed in the table. Participants were considered aware if they had been told by a health care professional that they had the condition.

c Calculated by using multivariate linear regression. Participants were considered treated if they reported currently taking prescribed medication for their condition.

d Estimates could not be calculated because of small sample sizes.

**Table 4:** Odds of Awareness, Treatment, and Control of Hypercholesterolemia Among Insured Adults Aged 20 to 64 Years With Hypercholesterolemia (n = 240), 2004 New York City Health and Nutrition Examination Survey^a^

Characteristic	Awareness,^b^ AOR (95% CI)	Treatment Among Aware,^b^ AOR (95% CI)	Total Cholesterol Among Aware^c^	

			β (SE)	*P* Value
**Routine place of care**				
No	0.1 (0-0.2)	0.1 (0-0.6)	33.8 (11.2)	.003
Yes	1 [Reference]		0	Reference
**Insurance type**				
Public	1.5 (0.7-3.4)	2.5 (1.0-6.4)	−11.8 (8.8)	.18
Private	1 [Reference]		0	Reference
**Age, y**				
20-39	0.2 (0.1-0.4)	0.1 (0-0.6)	35.1 (11.2)	.002
40-64	1 [Reference]		0	Reference
**Sex**				
Male	1.5 (0.7-3.0)	1.6 (0.7-3.6)	−7.3 (8.2)	.37
Female	1 [Reference]		0	Reference
**Race/ethnicity**				
Non-Hispanic white	1 [Reference]		0	Reference
Non-Hispanic black	0.8 (0.3-2.7)	1.2 (0.3-4.0)	6.3 (12.1)	.61
Non-Hispanic Asian	3.9 (1.1-13.7)	2.1 (0.5-9.3)	−22.3 (12.5)	.08
Hispanic	1.2 (0.4-3.4)	0.6 (0.2-1.9)	12.0 (11.7)	.30
**Country of birth**				
Foreign-born, in US <10 y	1.1 (0.4-3.1)	1.1 (0.3-4.0)	15.2 (14.5)	.29
Foreign-born, in US ≥10 y	0.7 (0.3-1.6)	0.7 (0.3-1.7)	5.3 (10.7)	.62
US-born	1 [Reference]		0	Reference
**Annual household income, $**				
<20,000	0.2 (0.1-0.7)	1.7 (0.6-4.8)	−6.0 (10.7)	.58
≥20,000	1 [Reference]		0	Reference
**Education level**				
<High school	1.7 (0.6-4.4)	1.2 (0.4-3.8)	3.4 (10.7)	.75
High school or equivalent	1.4 (0.6-3.5)	1.0 (0.3-3.1)	9.5 (9.6)	.32
>High school	1 [Reference]		0	Reference
**BMI, kg/m^2^**	1.1 (1.0-1.2)	1.0 (1.0-1.1)	−1.0 (0.6)	.10

Abbreviations: AOR, adjusted odds ratio; CI, confidence interval; SE, standard error; NC, not calculated; BMI, body mass index.

a All statistics were weighted.

b Calculated by using multivariate logistic regression adjusting for all covariates listed in the table. Participants were considered aware if they been told by a health care professional that they had the condition.

c Calculated by using multivariate linear regression. Participants were considered treated if they reported currently taking prescribed medication for their condition.
